# Does peanut butter transfer from hands to sports equipment?

**DOI:** 10.1186/1710-1492-7-S2-A5

**Published:** 2011-11-14

**Authors:** Sandy Kapur, Alisha Kapur, Wade Watson

**Affiliations:** 1 Department of Pediatrics, Dalhousie University, Division of Allergy, Halifax, Nova Scotia, B3K 6R8, Canada

## Background

Peanut is a robust allergen. Many parents are concerned about shared sports equipment for children with peanut allergy. Basketball is a common sport for many children, and involves frequent contact between the ball and hands. We assessed the potential for peanut butter to transfer from peanut contaminated hands to basketballs, and the potential to transfer back to hands.

## Methods

Baseline samples were taken from a previously used basketball and the hands of an avid basketball player. Five mL of peanut butter was applied to her hands and wiped with a commercial paper towel. The basket ball was dribbled for 5 minutes. Her hands were subsequently washed with regular soap and water. She then played with the potentially contaminated ball for another five minutes. Both rubber and leather basketballs were used. Samples from the ball and hands were taken at each step. The balls were cleaned with commercial bleach cleaner and resampled. Samples were analysed using a monoclonal-based Ara h 1 ELISA. The range of detection was 2-2000 ng/mL.

## Results

After application of peanut butter on hands, there was detectable peanut allergen. No detectable peanut was found on the surface of either basketball after 5 minutes of use. No detectable peanut was found on hands after 5 minutes of play with the potentially contaminated basketball.

## Conclusions

There is no significant transfer of peanut allergen from contaminated hands to basketballs. A peanut allergic individual is not at significant risk of an allergic reaction from playing basketball.

